# Supplementary figures and images for: Bacterial community structure of Anopheles hyrcanus group, Anopheles nivipes, Anopheles philippinensis, and Anopheles vagus from a malaria-endemic area in Thailand
Source: PLoS One. 2023 Aug 17;18(8):e0289733. doi: 10.1371/journal.pone.0289733 (PMC10434920; doi:10.1371/journal.pone.0289733)

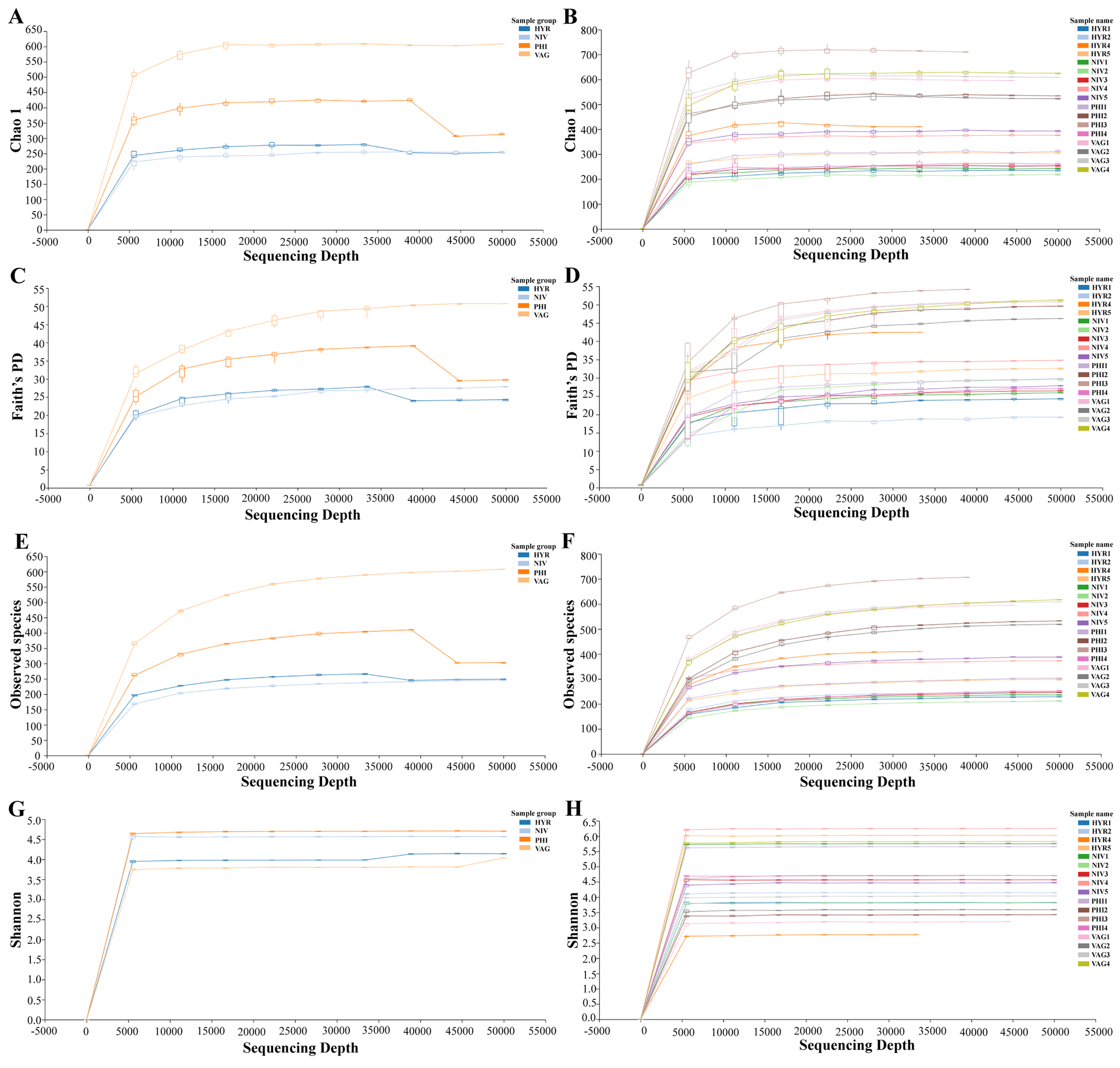

Supplement: S1 Fig — Rarefaction curves and depth of the richness and diversity indices included the Chao1 (A-B), Faith’s PD (C-D), observed species (E-F), and Shannon (G-H) of the microbial communities. A, C, E, and G represented data from each Anopheles group. B, D, F, and H represented data from each sample. (HYR: An. hyrcanus group; NIV: An. nivipes; PHI: An. philippinensis; VAG: An. vagus). (TIF) [file pone.0289733.s006.tif]
