# Supplementary material for: Bacterial community structure of Anopheles hyrcanus group, Anopheles nivipes, Anopheles philippinensis, and Anopheles vagus from a malaria-endemic area in Thailand
Source: PLoS One. 2023 Aug 17;18(8):e0289733. doi: 10.1371/journal.pone.0289733 (PMC10434920; doi:10.1371/journal.pone.0289733)
Supplement: Attachment — Submitted filename: Responses to Reviewers.docx [file pone.0289733.s007.docx]

**Point by point response to Journal Requirements, Editor and Reviewer comments**

**Journal Requirements:**

When submitting your revision, we need you to address these additional requirements.

1. Please ensure that your manuscript meets PLOS ONE's style requirements, including those for file naming. <https://journals.plos.org/plosone/s/file?id=wjVg/PLOSOne_formatting_sample_main_body.pdf> and <https://journals.plos.org/plosone/s/file?id=ba62/PLOSOne_formatting_sample_title_authors_affiliations.pdf>

**Reply:** We have done as suggested. We certified the submitted manuscript meets PLOS ONE's style requirements.

2. In your Methods section, please provide additional information regarding the permits you obtained for the work. Please ensure you have included the full name of the authority that approved the field site access and, if no permits were required, a brief statement explaining why.

**Reply:** No permits were required for the described study, which complied with all relevant regulations. All information and materials included in our manuscript are the original.

3. We note that you have stated that you will provide repository information for your data at acceptance. Should your manuscript be accepted for publication, we will hold it until you provide the relevant accession numbers or DOIs necessary to access your data. If you wish to make changes to your Data Availability statement, please describe these changes in your cover letter and we will update your Data Availability statement to reflect the information you provide.

**Reply:** We have done as suggested. All sequence reads have been deposited at the NCBI Sequence Read Archive (SRA) under project accession number PRJNA953178.Please see https://dataview.ncbi.nlm.nih.gov/object/PRJNA953178?reviewer=ieo2go5gsc7bs0jmhdog9a9r0v

4. We note that Figure 1 in your submission contain map/satellite images which may be copyrighted. All PLOS content is published under the Creative Commons Attribution License (CC BY 4.0), which means that the manuscript, images, and Supporting Information files will be freely available online, and any third party is permitted to access, download, copy, distribute, and use these materials in any way, even commercially, with proper attribution. For these reasons, we cannot publish previously copyrighted maps or satellite images created using proprietary data, such as Google software (Google Maps, Street View, and Earth). For more information, see our copyright guidelines: <http://journals.plos.org/plosone/s/licenses-and-copyright>.

We require you to either (a) present written permission from the copyright holder to publish these figures specifically under the CC BY 4.0 license, or (b) remove the figures from your submission:

a. You may seek permission from the original copyright holder of Figure 1 to publish the content specifically under the CC BY 4.0 license.

We recommend that you contact the original copyright holder with the Content Permission Form (<http://journals.plos.org/plosone/s/file?id=7c09/content-permission-form.pdf>) and the following text:

“I request permission for the open-access journal PLOS ONE to publish XXX under the Creative Commons Attribution License (CCAL) CC BY 4.0 (<http://creativecommons.org/licenses/by/4.0/>). Please be aware that this license allows unrestricted use and distribution, even commercially, by third parties. Please reply and provide explicit written permission to publish XXX under a CC BY license and complete the attached form.”

Please upload the completed Content Permission Form or other proof of granted permissions as an "Other" file with your submission.

In the figure caption of the copyrighted figure, please include the following text: “Reprinted from [ref] under a CC BY license, with permission from [name of publisher], original copyright [original copyright year].”

b. If you are unable to obtain permission from the original copyright holder to publish these figures under the CC BY 4.0 license or if the copyright holder’s requirements are incompatible with the CC BY 4.0 license, please either i) remove the figure or ii) supply a replacement figure that complies with the CC BY 4.0 license. Please check copyright information on all replacement figures and update the figure caption with source information. If applicable, please specify in the figure caption text when a figure is similar but not identical to the original image and is therefore for illustrative purposes only.

The following resources for replacing copyrighted map figures may be helpful: USGS National Map Viewer (public domain): <http://viewer.nationalmap.gov/viewer/>

The Gateway to Astronaut Photography of Earth (public domain):<http://eol.jsc.nasa.gov/sseop/clickmap/>

Maps at the CIA (public domain): <https://www.cia.gov/library/publications/the-world-factbook/index.html> and <https://www.cia.gov/library/publications/cia-maps-publications/index.html> NASA Earth Observatory (publicdomain): <http://earthobservatory.nasa.gov/>

Landsat: <http://landsat.visibleearth.nasa.gov/>

USGS EROS (Earth Resources Observatory and Science (EROS) Center) (public domain): [http://eros.usgs.gov/#](http://eros.usgs.gov/)

Natural Earth (public domain): <http://www.naturalearthdata.com/>

**Reply:** Figure 1 is original; no permits were required.

5. Please review your reference list to ensure that it is complete and correct. If you have cited papers that have been retracted, please include the rationale for doing so in the manuscript text or remove these references and replace them with relevant current references. Any changes to the reference list should be mentioned in the rebuttal letter that accompanies your revised manuscript. If you need to cite a retracted article, indicate the article’s retracted status in the References list and also include a citation and full reference for the retraction notice.

**Reply:** No retracted reference is included in the manuscript.

Additional Editor Comments: The manuscript needs a revision as recommended by the reviewers
**Reply:** We revised as the reviewer suggested.

**Reviewers' comments: Reviewer's Responses to Questions**

**Comments to the Author**

1. Is the manuscript technically sound, and do the data support the conclusions?
The manuscript must describe a technically sound piece of scientific research with data that supports the conclusions. Experiments must have been conducted rigorously, with appropriate controls, replication, and sample sizes. The conclusions must be drawn appropriately based on the data presented.

Reviewer #1: Partly

2. Has the statistical analysis been performed appropriately and rigorously?

Reviewer #1: N/A

3. Have the authors made all data underlying the findings in their manuscript fully available?

The [PLOS Data policy](http://www.plosone.org/static/policies.action#sharing) requires authors to make all data underlying the findings described in their manuscript fully available without restriction, with rare exception (please refer to the Data Availability Statement in the manuscript PDF file). The data should be provided as part of the manuscript or its supporting information, or deposited to a public repository. For example, in addition to summary statistics, the data points behind means, medians and variance measures should be available. If there are restrictions on publicly sharing data—e.g. participant privacy or use of data from a third party—those must be specified.

Reviewer #1: Yes

4. Is the manuscript presented in an intelligible fashion and written in standard English?

PLOS ONE does not copyedit accepted manuscripts, so the language in submitted articles must be clear, correct, and unambiguous. Any typographical or grammatical errors should be corrected at revision, so please note any specific errors here.

Reviewer #1: Yes

5. Review Comments to the Author

Please use the space provided to explain your answers to the questions above. You may also include additional comments for the author, including concerns about dual publication, research ethics, or publication ethics. (Please upload your review as an attachment if it exceeds 20,000 characters)

Reviewer #1: The manuscript by Wanchai Maleewong et al titled “Bacterial community structure of *Anopheles hyrcanus* group, *Anopheles nivipes*, *Anopheles philippinensis*, and *Anopheles vagus* from a malaria-endemic area in Thailand” is very important as it is focused on the investigation of the microbiota of some malaria vectors in Thailand. Despite the efforts put in place to control this deadly disease, it remains a public health mainly because of the resistance of mosquitoes to insecticides and the parasites themselves to drugs. Therefore, the use of microbiome based tools has been shown as an alternative vector control strategy. This work is then of great importance.

**Reply:** We would like to thank you for your kind suggestions and positive response. Your comments are encouraging and helpful.

However, some comments in order to improve the quality are as follows:

**Abstract**

In general, the authors need to add in the abstract the sample size and the software used for data analysis. Also, indicate if the DNA was extracted from the whole individual mosquito or the pool of mosquitoes, even the sequencing.

**Reply:** We modified as per your suggestion, “This study identified the bacterial microbiota in field-collected female adults of the *Anopheles hyrcanus* group and three *Anopheles* species*,* *Anopheles nivipes*, *Anopheles philippinensis*, and *Anopheles vagus*, from an endemic area in the southeastern part of Ubon Ratchathani Province, northeastern Thailand, near the Lao PDR-Cambodia-Thailand border. A total of 17 DNA libraries were generated from pooled female *Anopheles* abdomen samples (10 abdomens/sample). The mosquito microbiota was characterized through the analysis of DNA sequences from the V3−V4 regions of the 16S rRNA gene, and data were analyzed in QIIME2.” please see, page 2, lines 25−32.

In addition, the authors need to state the results of genetic diversity in terms of Alpha diversity and Beta diversity. Finally, it will be better to explain in one line the difference observed between these groups of bacteria.

**Reply:** We modified as per your suggestion, “Statistical difference in alpha diversity was observed between *An. hyrcanus* group and *An. vagus* and between *An. nivipes* and *An. vagus*, and beta diversity analyses showed that the bacterial community of *An. vagus* was the most dissimilar from other species.” please see, page 2, lines 34−36.

Line 31: “The results revealed the microbiota of four *Anopheles* species differed between pools of the same species and between species”. This result needs to be rephrased since *Anopheles* *hyrcanus* is stated here as a group and not a species.

**Reply:** We modified as per your suggestion, “A total of 3,442 bacterial ASVs were obtained, revealing differences in the microbiota both within the same species/group and between different species/group.” please see, page 2, lines 32−34.

Line 32: Before giving the predominant genus for each *Anopheles* groups, authors should give the predominant genus in the whole mosquito population of *An.* groups, even the total numbers of the identified bacterial. What about the predominant phylum? Also, the author should add the percentage to recover the overall abundance of these bacterial.

**Reply:** We modified as per your suggestion, “The most abundant bacteria belonged to the Proteobacteria phylum (48%-75%) in which *Pseudomonas*, *Serratia*, and *Pantoea* were predominant genera among four *Anopheles* species/group. However, the most significantly abundant genus observed in each *Anopheles* species/group was as follows: *Staphylococcus* in the *An. hyrcanus* group, *Pantoea* in the *An. nivipes*, *Rosenbergiella* in *An. philippinensis*, and *Pseudomonas* in *An. vagus*. Particularly, *Pseudomonas* sp. was highly abundant in all *Anopheles* species except *An. nivipes*.” please see, page 2, lines 36−42.

Line 38: “The present study provides new knowledge on the microbiota of four potential malaria vectors as a starting step towards understanding ….”. In the discussion, it is stated that it is the first study done in this context the authors should rewrite this sentence in the abstract such that it clearly appears that it is the first study and not only new knowledge as they said.

**Reply:** We changed “new knowledge” to “first study” as per your suggestion. “The present study provides the first study on the microbiota of four potential malaria vectors as a starting step towards understanding the role of the microbiota on mosquito biology and ultimately the development of potential tools for malaria control. Please see, page 2, lines 42−45.

**Methods**

Line 99: The authors said, “It is very difficult to identify species within the *An. hyrcanus* group based on morphology alone”. What about molecular identification? The authors should check if there is no protocol for that.

**Reply:** *Anopheles hyrcanus* group was used based on morphology alone (reference no. 33; Mosquito Taxonomic Inventory. *Anopheles* classification. Available from: https://mosquito-taxonomic-inventory.myspecies.info/node/11358.), until now, no accurate molecular identification is reported.

Line 100: “For that reason, we used our specimens belonging to this species group as *An. hyrcanus* group”, this sentence is not comprehensible and cannot be linked to the previous. Rewrite it to let it make sense.

**Reply:** We changed to “It is very difficult to identify species within the *An. hyrcanus* group based on morphology alone [33]. Then, we termed our specimens belonging to *An. hyrcanus* group. Please see, page 5, lines 104−106.

Line 102: The authors said, “Female *Anopheles* mosquitoes were dissected for detection and identification of *Plasmodium* parasites”. The parts dissected should be cited, are they abdomens, Head and thorax or ovaries…? Also, the method used to identify *Plasmodium* needs to state.

**Reply:** We modified to “The head and thorax were separated from the abdomen using sterile forceps for detection of *Plasmodium* parasites by molecular method [34].” Please see, page 5, lines 106−107.

Line 104: “Ten abdomens of each *Anopheles* species collected on the same day at around the same time were pooled …. bacterial community structure”. How many abdomens one pool? How many pools have been made for each species? These must be clear in the sentence. Also, add the estimated days collections.

**Reply:** We modified as per your suggestion, “Ten abdomens of each *Anopheles* species/group collected on the same day at around the same time were pooled per sample; *An. hyrcanus* group (HYR; *n* = 4 pools), *An. nivipes* (NIV; *n* = 5 pools), *An. philippinensis* (PHI; *n* = 4 pools), and *An. vagus* (VAG; *n* = 4 pools) and kept at -20 °C until DNA extraction for further investigation of malaria and bacterial community structure.” Please see, pages 5−6, lines 109−113.

Line 115: “The presence of *Plasmodium* spp. parasites was investigated based on partial cox1 gene amplification”. The authors should well state the part of the mosquito used here for *Plasmodium* detection.

**Reply:** We modified to “The presence of *Plasmodium* spp. DNA was investigated based on partial *cox1* gene amplification using DNA samples extracted from separated abdomen specimens.” Please see, page 6, lines 122−123.

Line 126: “From laboratory *Plasmodium falciparum* and *Plasmodium vivax* in-vitro cultures was used as 127 positive controls”: Why not used *Plasmodium* spp. as a positive control?

**Reply:** Now, no other laboratory *Plasmodium* spp. were available in our systems. However, COX-IF (5’ -AGA ACG AAC GCT TTT AAC GCC TG-3’and COX-IR (3’-ACT TAA TGG TGG ATA TAA AGT CCA TCC wGT-5’) primers are the conserve primers that can amplified other *Plasmodium* spp (*Plasmodium falciparum*, *Plasmodium vivax*, *Plasmodium knowlesi*, *Plasmodium malariae*, *Plasmodium ovale wallikeri* and *Plasmodium ovale curtisi*). Reference no, 34 (Echeverry et al. 2017).

Line 130: “The universal region-specific primers 341F (5’-CCT AYG GGR BGC ASC AG-3’) and 806R (5’-GGA CTA CNN GGG TAT CTA AT-3’…V3-V4 regions of the 16S rRNA gene were used.” Reference needs to be added here.

**Reply:** We added as per your suggestion, “The universal region-specific primers 341F (5’-CCT AYG GGR BGC ASC AG-3’) and 806R (5’-GGA CTA CNN GGG TAT CTA AT-3’) (NovogeneAIT Genomics Pte. Ltd., Singapore) tagged with sample-identifying barcodes to amplify the V3−V4 regions of the 16S rRNA gene were used.” Please see, page 7, lines 139−142.

Line 129: “Bacterial 16S rRNA gene amplification and sequencing”. In this paragraph, the authors should clearly give the details on the numbers of samples used, which parts have been used, which *Anopheles* species and how many pools?

**Reply:** We modified to “Seventeen DNA samples from pools of ten abdomens of each *Anopheles* species: *An. hyrcanus* group (HYR; *n* = 4 pools), *An. nivipes* (NIV; *n* = 5 pools), *An. philippinensis* (PHI; *n* = 4 pools), and *An. vagus* (VAG; *n* = 4 pools) were used for amplification and sequencing.” Please see, page 7, lines 137−139.

Line 134-138: The authors need to describe in detail the methodology for sample sequencing. Some previous studies have more describe this part. Also indicated whether the negative and positive controls have been used during the sequencing.

**Reply:** In the Illumina sequencing platform, quality control is carried out at each step of the process from DNA samples to the results, including sample testing, PCR, purification, library construction, and sequencing (no requirement for negative or positive control). We have added more information about quality control in the method: “At each step of the process, quality control was carried out to maintain the accuracy and reliability of the sequencing data according to the Illumina sequencing system (NovogeneAIT Genomics).” Please see, page 7, lines 145−147.

**Results**

In general, the authors need to indicate which season the mosquitoes have been collected, because some previous study show that the season could have a great impact on microbiota diversity.

**Reply:** We added sentence “covering three seasons (hot, rainy, and cold)” as per your suggestion, “The mosquitoes were collected throughout the study period, covering three seasons (hot, rainy, and cold).” Please see materials and methods section, page 5, lines 93−95.

Line 163: Before talking about the results on the bacterial composition, authors should first give the results of mosquitoes identification. It has been stated in the methodology that the four species used were the more predominant, what about the about? And which are the frequencies?

**Reply:** Results of mosquito identification and species composition were added as suggested,

“A total of 2,659 *Anopheles* females belonging to one group (*An. hyrcanus* group) in the subgenus *Anopheles* and nine species/group (*An. dirus* s.l., *An. karwari*, *An. kochi*, *An. maculatus* group, *An. minimus* s.l., *An. nivipes*, *An. philippinensis*, *An. tessellatus* and *An. vagus*) in the subgenus *Cellia* were collected throughout study period. The four most abundant taxa were *An. hyrcanus* group (78.15%) followed by *An. philippinensis* (14.70%), *An. nivipes* (5.00%) and *An. vagus* (0.90%).” Please see, pages 8−9, lines 178−183.

Line 164. In this paragraph, the authors should mention the quality control statistic: the number of raw reads obtained, the number obtained after filtration, the rarefication…

**Reply:** We were represented in S1 Table, please see S1 Table. For clearer, we added “The output statistics are shown in S1 Table.” Please see, page 9, lines 186−187.

Line 180: “The microbiota of four *Anopheles* species differed between pools of the same species and between species”. Here the authors mentioned a difference between the pools of the same species, whereas the title on line 179 only mentions the differences between species. Moreover, in the following sentences, the differences they show are only those between species. What are about the differences between the same species?

**Reply:** Thank for your clarification, for better understanding, we modified and added more data to the S5 Table, and the added sentences “Shared ASVs were dominant bacteria and a small number of ASVs were different between samples in the same *Anopheles* species/group (S5 Table).” and “The microbiota of four *Anopheles* species/group were differed between the same and between species/group (S4 Table and S5 Table).” Please see S4 Table and S5 Table and revised text, page 9, lines 193−194 and lines 200−201.

Line 187: “The Pseudomonadaceae was dominant in *An. vagas* and *An. hyrcanus* group, whereas …Carnobacteriaceae and Burkholderiaceae were abundant in *An. hyrcanus* group and *An. vagas*, respectively (Fig 3B). This paragraph should be complete, the authors mentioned the predominance in the 4 groups of mosquitoes but for the abundance, they just referred to *An. vagas* and *An. hyrcanus*, what about the 2 others? Also, to what the predominance and abundance here referred?

**Reply:** We have added already, please see the sentences “The Pseudomonadaceae was dominant in *An. vagas* and *An. hyrcanus* group, whereas Erwiniaceae was dominant in *An. philippinensis* and *An. nivipes*, followed by Moraxellaceae. Carnobacteriaceae and Burkholderiaceae were abundant in *An. hyrcanus* group and *An. vagas*, respectively (Fig 3B).” Please see, page 10, lines 207−210.

Line 205: “*Elizabethkingia meningoseptica*, *Asaia krungthepensis*, …species except *An. nivipes*”. Is this statement, are the authors sure that *Asaia* that they found where specifically *krungthepensis*? If not, they should better write *Asaia* sp.

**Reply:** Yes, we did discover the *Asaia krungthepensis* species that AVS named "OTU 13". Please see S2 Table and Fig. 4B. We also double-checked using BLASTn, and sequences were found corresponding to the species of *Asaia krungthepensis* (100% similarity).

**Discussion**

Line 238: “However, information on the gut microbiota of these four *Anopheles* species, which was characterized for further study when positive malaria samples are available for comparison”. This sentence must be rewritten; something is missing.

**Reply:** We modified as per your suggestion. “However, the limit information on the gut microbiota of these four *Anopheles* species/group were obtained, which was required for further study when positive malaria samples are available for comparison. Please see, page 12, lines 259−261.

Line 285: “The genus *Rosenbergiella* was the most abundant taxon in *An. philippinensis*”. It is just during the discussion that information is clear while it in the results, it is just noticed that this genus is among the most abundant. Since the idea is discussed, this part should be rephrased in the result such the reader is not surprised at the part discussion.

**Reply:** We modified as per your suggestion in results section. “For *An. philippinensis*, *Rosenbergiella* was the highest significant, followed by *Bacillus, Acinetobacter,* and *Romboutsia.*” Please see, page 10, lines 218−220.

Line 294: “Significantly, *Pseudomonas aeruginosa* and *Carnobacterium maltaromaticum* were found to be dominant in the guts of female *An. hyrcanus* group”. The authors should continue to use the term abdomen rather than gut, there is a slide difference.

**Reply:** We changed “guts” to “abdomens” as per your suggestion. “Significantly, *Pseudomonas aeruginosa* and *Carnobacterium maltaromaticum* were found to be dominant in the abdomens of female *An. hyrcanus* group.” Please see, page 14, lines 315−316.

Finally, we appreciate the reviewer very much for your kind suggestions, your comments are supportive and helpful.
