# Supplementary material for: Bacterial community structure of Anopheles hyrcanus group, Anopheles nivipes, Anopheles philippinensis, and Anopheles vagus from a malaria-endemic area in Thailand
Source: PLoS One. 2023 Aug 17;18(8):e0289733. doi: 10.1371/journal.pone.0289733 (PMC10434920; doi:10.1371/journal.pone.0289733)
Supplement: Attachment — Submitted filename: Responses to Reviewers 16-7-23.docx [file pone.0289733.s008.docx]

**Point by point response to Editor and Reviewer comments.**

ACADEMIC EDITOR: The manuscript needs revisions according to the reviewers opinion.

Reply: we modified as the reviewer comment.

Reviewers' comments: Reviewer's Responses to Questions

**Comments to the Author**

6. Review Comments to the Author

Reviewer #1: The authors need to more describe in detail the bioinformatics analysis. Some previous studies have more describe this part. To establish whether alpha diversity differs across mosquito species for all samples, the authors need to consider the rarification depth (add this figure in supplementary data) of ASVs per sample, which will be sufficient to capture the typical low microbiota diversity in individual mosquitoes. You can see the link of the paper published recently: <https://www.biorxiv.org/content/10.1101/2023.06.23.546313v1>.

**Reply:** We added more data to the S1 Figure and added the sentences, “Moreover, the abundance of ASVs was used to generate a rarefaction curve for estimating the species/group richness and diversity in the microbiota of four *Anopheles* species/group.” and “The alpha rarefaction curve represents the sequence to a sufficient depth of the sample, with most reaching saturation, which was sufficient to capture the scope of microbial diversity and indirectly reflect the abundances of our *Anopheles* species/group (S1 Fig).” as per your suggestion, Please see S1 Figure and revised text, page 8, lines 168–170, page 9, lines 191–194, and page 25, lines 557–562.

The S1 Figure did not include ACE and Simpson indices. For better understanding, the ACE index means the measuring of bacterial community richness can be also represented in the observed species and Chao1 indices. While Simpson index means bacterial diversity can also be represented in the Shannon index.

Finally, we would like to thank the Academic Editor and Reviewer for the supportive and helpful comments. We hope our revised manuscript would give you satisfaction.
